# Messages that increase women’s intentions to abstain from alcohol during pregnancy: results from quantitative testing of advertising concepts

**DOI:** 10.1186/1471-2458-14-30

**Published:** 2014-01-13

**Authors:** Kathryn E France, Robert J Donovan, Carol Bower, Elizabeth J Elliott, Janet M Payne, Heather D’Antoine, Anne E Bartu

**Affiliations:** 1 School of Marketing, Tourism and Leisure, Edith Cowan University, 270 Joondalup Drive, Joondalup, Western Australia 6027, Australia; 2 Centre for Behavioural Research in Cancer Control, Curtin University, GPO Box U1987, Perth, Western Australia 6845, Australia; 3 Telethon Institute for Child Health Research, Centre for Child Health Research, The University of Western Australia, PO Box 855, West Perth, Western Australia 6872, Australia; 4 Discipline of Paediatrics and Child Health, Sydney Medical School, The University of Sydney, The Children’s Hospital at Westmead, Locked Bag 4001, Westmead, NSW 2145, Australia; 5 School of Nursing and Midwifery, Faculty Health Sciences, Curtin Health Innovation Research Institute, Curtin University, GPO Box U1987, Perth, Western Australia 6845, Australia

**Keywords:** Alcohol, Advertising, Pregnancy, Messages, Campaigns, Threat, Self-efficacy, Fear, FASD

## Abstract

**Background:**

Public awareness-raising campaigns targeting alcohol use during pregnancy are an important part of preventing prenatal alcohol exposure and Fetal Alcohol Spectrum Disorder. Despite this, there is little evidence on what specific elements contribute to campaign message effectiveness. This research evaluated three different advertising concepts addressing alcohol and pregnancy: a threat appeal, a positive appeal promoting a self-efficacy message, and a concept that combined the two appeals. The primary aim was to determine the effectiveness of these concepts in increasing women’s intentions to abstain from alcohol during pregnancy.

**Methods:**

Women of childbearing age and pregnant women residing in Perth, Western Australia participated in a computer-based questionnaire where they viewed either a control or one of the three experimental concepts. Following exposure, participants’ intentions to abstain from and reduce alcohol intake during pregnancy were measured. Other measures assessed included perceived main message, message diagnostics, and potential to promote defensive responses or unintended consequences.

**Results:**

The concepts containing a threat appeal were significantly more effective at increasing women’s intentions to abstain from alcohol during pregnancy than the self-efficacy message and the control. The concept that combined threat and self-efficacy is recommended for development as part of a mass-media campaign as it has good persuasive potential, provides a balance of positive and negative emotional responses, and is unlikely to result in defensive or unintended consequences.

**Conclusions:**

This study provides important insights into the components that enhance the persuasiveness and effectiveness of messages aimed at preventing prenatal alcohol exposure. The recommended concept has good potential for use in a future campaign aimed at promoting women’s intentions to abstain from alcohol during pregnancy.

## Background

Alcohol is a teratogen and consumption during pregnancy can result in a range of conditions collectively referred to as the Fetal Alcohol Spectrum Disorders (FASD). In Australia, though the prevalence of FASD is unknown, the last decade has seen a substantial rise in the profile of alcohol use during pregnancy as a public health issue [1] and the current national guidelines for alcohol use recommend that for pregnant women, “not drinking is the safest option” [2]. At a population level, the promotion of this abstinence-based message is fraught with challenges. First, the risk of poor outcomes resulting from prenatal alcohol exposure is inconclusive, particularly in relation to low levels of exposure [3]. In this climate, risk communication lacks specificity and the rationale for abstinence lacks certainty. Related to this, recommendations and public policy regarding alcohol use during pregnancy have been inconsistent between and within countries [4,5] and likely to have contributed to confusion amongst women with regard to alcohol use during pregnancy [6]. While abstinence-based recommendations are increasing internationally [4,7,8], these too have come under scrutiny for the way in which uncertainty is positioned to support a risk-avoidance approach to behaviour [9-11] and for being “paternalistic” [12]. Thus, a key challenge in providing advice through mass media for pregnant women to avoid alcohol is to create messages that are persuasive, credible and evidence-based, and that do not exacerbate confusion. Furthermore, with the potential for abstinence-based messages to contribute to feelings of worry, guilt and shame amongst women who cannot consider abstinence or who have consumed alcohol before they knew that they were pregnant, a further challenge is to create messages that motivate behaviour change but do not result in defensive or maladaptive responses amongst message recipients [13].

In seeking to create persuasive communications and instigate changes in behavioural intentions, it is important to determine which constructs and processes are relevant to the target audience, are predictive of the behaviour in question, and can be influenced to promote the desired behaviour change [14]. Although many campaigns targeting alcohol use during pregnancy have been conducted, predominantly in North America, few have reported formative testing or evaluation [15-17]. Thus, there is a lack of evidence to inform the design of effective campaign strategies and messages. To address this, formative research was conducted with pregnant women and women of childbearing age in Western Australia to develop advertising concepts to increase women’s intentions to abstain from alcohol during pregnancy [18]. This research resulted in three concepts based on two overall approaches: a threat approach based on fear and worry, and a positive approach that sought to promote self-efficacy. This paper outlines the testing of these three concepts to confirm their effectiveness (or otherwise) in increasing women’s intentions and confidence to abstain from alcohol use during pregnancy. Given the sensitive and complex nature of the issue, it was considered essential that message strategies also be tested for potential defensive or unintended effects.

Two threat concepts and one positive concept based on self-efficacy were tested against a control concept. Participants’ responses to one of the four concepts (shown in story-board format) were measured through a computer-based survey. This formative development and testing was considered the first step for creating persuasive, population-based messaging for alcohol use during pregnancy, particularly given the lack of evidence around effective message strategies. Furthermore, given the sensitive and complex nature of the issue, it was important that message strategies were tested for potential defensive or unintended effects.

As noted by Cismaru and colleagues [19], many campaigns have adopted a threat-based message and have focused on convincing women that the consequences of alcohol use during pregnancy are severe. However the concept of self-efficacy, or confidence in one’s ability to a adopt behaviour change (which is a critical component of models describing the influence of a threat on behaviour change) is frequently overlooked [19]. As such, we specifically sought to investigate the effectiveness of a positive (self-efficacy based) appeal, compared to a straight threat appeal and one that combined the two.

The objectives of the research were to:

1) Confirm the effectiveness of the concepts in increasing women’s intentions and confidence to abstain from alcohol use during pregnancy;

2) Compare the relative effectiveness of the concepts in increasing women’s intentions and confidence to abstain from alcohol use during pregnancy; and

3) Assess whether and to what extent any of the three experimental concepts prompted defensive responses or unintended effects amongst the message recipients.

## Methods

### Design

Two threat concepts and one positive concept based on self-efficacy were tested against a control concept. Testing of the concepts was conducted through a computer-based questionnaire that measured participants’ responses following exposure to the control or one of the three experimental concepts. After answering a series of screening questions to ensure the participants were eligible, participants were shown a concept on the screen in story-board format. All participants were randomly assigned to the conditions except for those women who received an emailed questionnaire link and identified themselves as pregnant. For ethical reasons, these women were automatically assigned to the control concept to ensure that they would not be exposed to an advertisement about alcohol and pregnancy without a comprehensive informed consent and duty-of-care process. Pregnant participants who saw one of the experimental concepts were recruited through a different process (described below).

**Figure 1:**
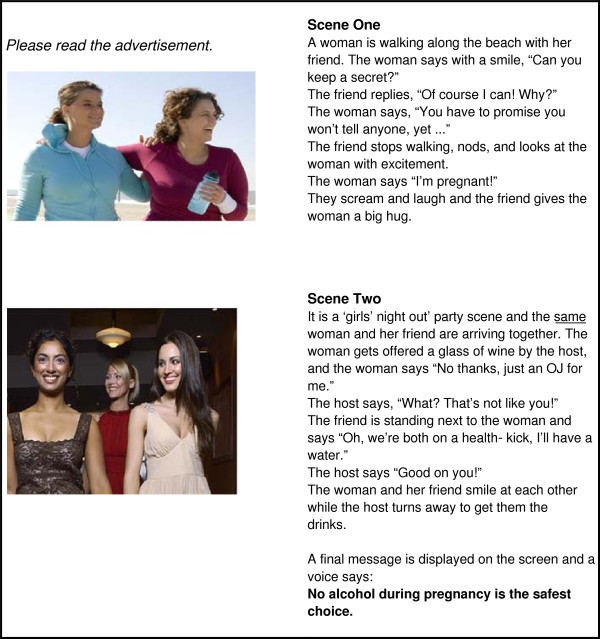
Self-efficacy only.

**Figure 2:**
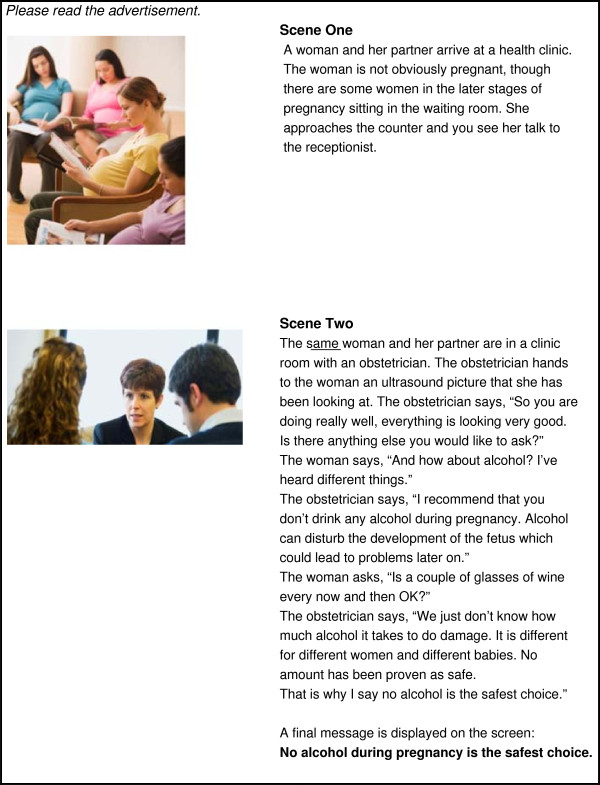
Threat only.

**Figure 3:**
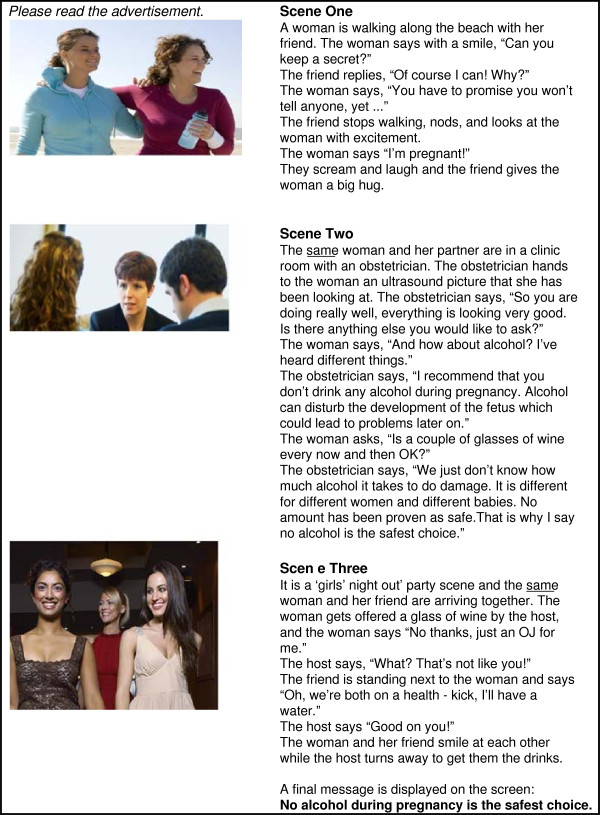
Threat and self-efficacy.

**Figure 4:**
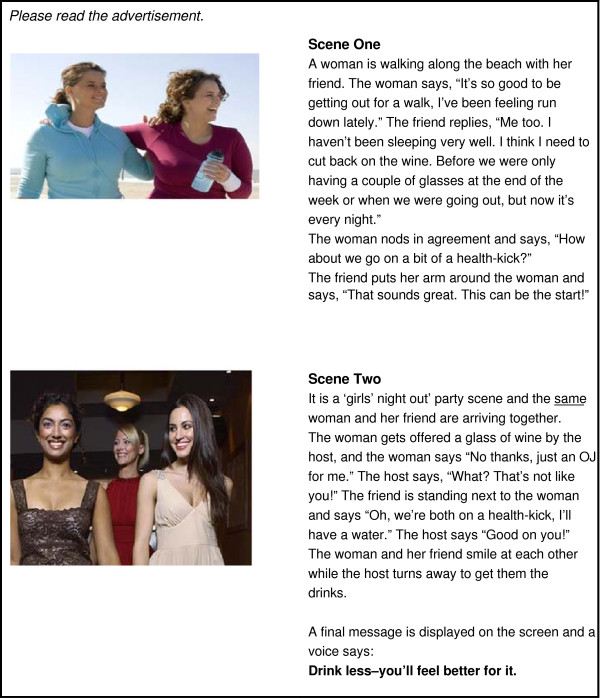
**Control** ***.***

The three experimental concepts and the control concept are shown in Figures 1, 2, 3, 4 and are named as per their construct composition, namely;

1) *Self-efficacy only*

2) *Threat only*

3) *Threat and self-efficacy*

4) *Control*

*Self-efficacy only* (Figure 1) depicts a woman confiding in her friend that she is pregnant. The friend then supports the woman to avoid alcohol by abstaining from alcohol herself at a social function and using the excuse of being on a ‘health-kick’ in order to avoid unwanted disclosure or questions about pregnancy. This concept focuses on modelling positive behaviour and social belonging and acceptance. It promotes a sense of self-efficacy in support of behaviour change by using the influence of a significant other to support a woman’s abstinence from alcohol during pregnancy. The concept promotes a ‘no alcohol in pregnancy is the safest choice’ message. *Threat only* (Figure 2) depicts a woman in the early stages of pregnancy in a clinical setting. The woman asks her obstetrician about alcohol, and the obstetrician provides information and advice regarding alcohol use during pregnancy. The concept focuses on a generalised threat and risk of alcohol use during pregnancy as the motivation for behaviour change. It promotes a message that ‘no alcohol in pregnancy is the safest choice’. *Threat and self-efficacy* (Figure 3) combines the scenarios of *threat only* and s*elf-efficacy* as an interchanging series of scenes. *Control* (Figure 4) is a ‘drink less’ advertising concept that does not include any reference to, or information about, the period of pregnancy, nor any reference to specific negative consequences of alcohol consumption. The control promotes a ‘drink less – you’ll feel better for it’ message.

### Participants

Given the difficulties of achieving a large sample of pregnant women, participants were largely women of childbearing age who were not pregnant (n = 354). However we also recruited a smaller sample of pregnant women (n = 116) to identify any substantial differences between this group and the sample of non-pregnant women that may indicate a need for further concept development and testing with pregnant women. Non-pregnant participants were recruited using a variety of methods. First, women who had in previous research indicated their interest in participating in research on women’s health were invited to participate through an email containing a link to the questionnaire. These participants were given the option to forward the link to friends. A market research company was also contracted to send the questionnaire link to eligible panel members. Consent to participate was implied by accessing the questionnaire through the online link. Non-pregnant women were eligible to participate if they were aged 18–45 years (inclusive), were residing in Perth, Western Australia and currently drank alcohol. Pregnant women were recruited from antenatal classes and clinical care venues, and were eligible to participate even if they nominated that they did not currently drink alcohol. Written, informed consent was obtained from these participants.

All participants were randomly assigned to either the control or one of the experimental conditions, except for those women who received an emailed questionnaire link and were pregnant. For ethical reasons, these women were automatically assigned to the control concept to ensure that they would not be exposed to an advertisement about alcohol and pregnancy without a comprehensive informed consent and duty-of-care process, such as that provided through the process employed within the antenatal settings.

### Measures and analysis

The primary measures of concept effectiveness were:

• Women’s intentions to try to abstain from alcohol in a future (non-pregnant sample) or current (pregnant sample) pregnancy;

• Women’s intentions to try to reduce their alcohol intake in a future (non-pregnant sample) or current (pregnant sample) pregnancy;

• Women’s confidence that they could try to abstain from alcohol in a future (non-pregnant sample) or current (pregnant sample) pregnancy; and

• Women’s confidence that they could try to reduce their alcohol intake in a future (non-pregnant sample) or current (pregnant sample) pregnancy.

Intentions and confidence were measured by single-item questions that were measured along a five-point scale. The mean responses were calculated along with the percentage of participants who nominated the highest level of intent or confidence (referred to as the ‘top box’ response). For example, for the question, *Having read the advertisement, are you more or less likely to try to stop drinking alcohol completely during a future pregnancy?* the response options and their respective values were:

• Much more likely to try to stop (1) - (t.b. = ‘top box’)

• More likely to try to stop (2)

• No different (3)

• Less likely to try to stop (4)

• Much less likely to try to stop (5)

In this example, the ‘top box’ response is “Much more likely to try to stop” and the measure is the percentage of participants who nominated this box (% t.b). Donovan and Henley [14] and Urban and colleagues [20] note that within consumer research, different weightings have been established for these likelihood scales based on their ability to predict actual purchase. That is, simply analysing the mean response is likely to underestimate the extent of the relative impact of the concept on intentions. Hence, ‘top box’ percentage comparisons provide a better indication of the relative impact of the concepts with respect to translating intentions into behaviour.

The questionnaire also collected demographic data, such as age and socioeconomic status, and personal profile data such as frequency of alcohol use and at-risk alcohol consumption (as measured by AUDIT-C [21]). Women’s responses to the concepts were cross-tabulated by several of these demographic and personal characteristics to determine if different women were responding differently to the concepts, and to highlight any potential audience segments. Women’s responses to the concepts were analysed by frequency of alcohol consumption, socioeconomic status, education level and whether or not they had children.

To confirm the presence or absence of threat and self-efficacy in the concepts, participants were asked whether the advertisement implied or suggested that:

• If you were pregnant and trying to stop drinking alcohol during social situations it could be easier than you thought; and

• If you drank alcohol during pregnancy the impact on the unborn baby could be mild, moderate or severe.

Participants could also nominate that the advertisement did not imply or suggest these at all. Presence of self-efficacy was measured by the percentage of respondents who nominated that the advertisement implied that it was easier to stop drinking alcohol during social situations. Threat was measured by the percentage of participants who nominated that the advertisement suggested that there could be a mild, moderate or severe effect on the unborn baby.

Message factors influencing effectiveness, such as perceived main messages, believability and relevance were assessed, along with several message diagnostics such as likeability, novelty, and appeal in terms of being interesting, convincing and providing important information. These factors were measured using open-ended (e.g. “What do you think is the main message in this advertisement? What is it telling people?”) and close-ended single-item questions (e.g. “How believable did you find the advertisement” with the response options: “very believable”, “quite believable”, “a little believable”, “not believable” and “not at all believable”). Participants were also asked if they had “any of the following feelings while reading the advertisement”: worried, anxious, guilty, regretful, ashamed, surprised, relieved, proud of myself, or happy.

To determine the likelihood of the concepts resulting in defensive responses participants were asked if they had any of the following thoughts while they were reading the advertisement: “I don’t want to think about what the advertisement is saying”; “the information in this advertisement is false”; “the advertisement exaggerates the issue”; and “the advertisement is misleading”. Dichotomous response data such as these (yes or no) were analysed by the percentage of participants who nominated the affirmative response (yes %). Differences were explored using Chi-Square testing. Finally, to measure the potential for the concepts to promote unintended counterproductive responses, participants were given a range of options and asked whether they would do any of these if they had drunk alcohol in the early stages before they knew they were pregnant. These options included “I would talk to a health professional about options for terminating a pregnancy” and “I would drink the same amount of alcohol as usual for the rest of the pregnancy”.

### Ethics approval

The research was approved by the Edith Cowan University Human Research Ethics Committee. Approval to conduct the research with pregnant women was also received by the Government of Western Australia, Department of Health, Women and Newborns Health Service Ethics Committee; Government of Western Australia, Department of Health, South Metropolitan Allied Health Service Human Research Ethics Committee; and Mercycare Ethics Committee.

## Results

Given the relatively small numbers of pregnant women assigned to each exposure condition (n = 40 to *Control*; n = 25/26 to each of the three concepts), the results for pregnant women are not presented in detail here. Furthermore, as some questions differed slightly for pregnant and non-pregnant women, only results from the larger non-pregnant sample are reported. Overall, no substantial differences in relative reactions that would warrant concept modification were identified between the pregnant and non-pregnant women.

### Participants

The demographic characteristics of the non-pregnant participants are shown in Table 1. There was a relatively even spread of age groups, but a bias towards post school qualifications, and only just over a quarter living in lower SES postcodes. Just over half had at least one child, with the majority of those having two or more children. Approximately one in five had never married and almost one in three thought they might become pregnant in the next two years. With respect to alcohol consumption, approximately seven in ten scored positive for alcohol misuse (with a composite score of ≥3 on the AUDIT-C alcohol screening tool [21]), and just under half drank two or more times a week, with a similar proportion drinking three or more drinks on a typical drinking occasion. Table 2 shows the number of non-pregnant participants who saw each concept.

**Table 1:** Demographic characteristics (n = 354)

**Demographic measure**	**Participants (n = 354) n (%)**
*Age*	
18-24 yrs	53 (15.0)
25-29 yrs	85 (24.0)
30-34 yrs	71 (20.1)
35-39 yrs	85 (24.0)
40-45 yrs	60 (16.9)
*Education – highest level*	
Year 9 or below	2 ( 0.6)
Year 10 or 11	34 ( 9.6)
Year 12	66 (18.6)
Trade certificate	24 ( 6.8)
Non-trade certificate	33 ( 9.3)
Associate/undergrad diploma	40 (11.3)
Bachelor degree	98 (27.7)
Post-graduate degree	57 (16.1)
*Marital status*	
Never married	79 (22.3)
Widowed	2 ( 0.6)
Divorced	15 ( 4.2)
Separated	10 ( 2.8)
Married/de-facto	248 (70.1)
Socio-economic status*	
Live in suburb ≤ 50%	96 (27.2)
Live in suburb 51–84%	137 (38.6)
Live in suburb ≥ 85%	121 (34.2)
*Children*	
Have children	190 (53.7)
Do not have children	164 (46.3)
*Parity - those with children:*	
1 child	64 (33.7)
2 children	84 (44.2)
3 children	31 (16.3)
4 children	7 ( 3.7)
5 or more	4 ( 2.1)
*Might become pregnant in next 2 years*	
Yes	110 (31.1)
No	168 (47.5)
Unsure	76 (21.5)
*Alcohol – positive score for alcohol misuse*	
(AUDIT-C – a composite score ≥3)	260 (73.4)
*Alcohol - how often have a drink containing alcohol*	
4 or more times/week	51 (14.4)
2-3 times/week	111 (31.4)
2-4 times/month	118 (33.3)
Monthly or less	74 (20.9)
Never	N/A (not eligible)
*Alcohol - how many drinks on a typical occasion*	
10 or more	7 ( 2.0)
7, 8 or 9	15 ( 4.2)
5 or 6	44 (12.4)
3 or 4	85 (24.0)
1 or 2	203 (57.3)

*Measured by suburb of residence according to state percentiles of advantage/disadvantage [22].

**Table 2:** Number of participants exposed to each concept

			**Concept**	
	***Control***	***Self-efficacy only***	***Threat only***	***Threat and self-efficacy***
**Non-pregnant**	*58*	*132*	*83*	*81*
**Pregnant**	*40*	*26*	*25*	*25*
**TOTAL**	*98*	*158*	*108*	*106*

### Analyses

Significant differences in ‘top box’ percentages for each concept were explored using Chi-Square testing. Analysis of variance and the Scheffe post-hoc tests [23] were conducted to determine whether there were any significant differences between the mean responses. Significance at the value of p ≤ 0.05 is indicated in the results tables with bold type and symbols (key shown below each table). Given the applied aims of the study, we focus primarily on the ‘top box’ analyses in this paper. As anticipated, the Tables indicate that many variables that were significant via a ‘top box’ analysis showed no significant difference on mean scores – thus ‘masking’ differences in impact of the different concepts.

### Presence of threat and self-efficacy within the concepts

The presence of threat in the concepts was confirmed: 74.7% of women who saw *Threat only* and 82.7% who saw *Threat and self-efficacy* perceived a threat in the message compared with 41.7% of participants who saw *Self-efficacy*. Self-efficacy was also confirmed: 85.6% of women who saw *Self-efficacy only* and 75.3% who saw *Threat and self-efficacy* perceived that the advertisements implied that reducing or stopping drinking during a social situation could be easier than they thought. In contrast, only 12.0% of women who saw *Threat only* perceived this self-efficacy message.

### Intentions and confidence to abstain from and reduce alcohol during pregnancy

All three experimental concepts significantly increased women’s intention to abstain from alcohol in a future pregnancy, and the two threat concepts also increased women’s intention to reduce alcohol use in a future pregnancy, compared to the control (Table 3). The two threat concepts increased women’s confidence to reduce alcohol during pregnancy compared to the control, but *Self-efficacy only* did not.

**Table 3:** Behavioural intentions and confidence to modify behaviour by each concept (n = 354)

	***Control***	***Self-efficacy only***	***Threat only***	***Threat and self-efficacy***
	**n = 58**	**n = 132**	**n = 83**	**n = 81**
**Measure**	**t.b. %**$\bar{x}$	**t.b. %**$\bar{x}$	**t.b. %**$\bar{x}$	**t.b. %**$\bar{x}$
Intentions to try to ABSTAIN in future pregnancy	19.0 2.62	29.5^#^ 2.23	48.2^#^^ 1.88*	48.1^#^^ 1.99*
Intentions to try to REDUCE in future pregnancy	17.2 2.52	23.5 2.3	36.1^#^^ 1.9*	44.4^#^^ 2.02
CONFIDENCE to ABSTAIN in a future pregnancy	29.5 2.40	27.3 2.24	42.2^#^^ 2.04	44.4^#^^ 1.99
CONFIDENCE to REDUCE in a future pregnancy	20.7 2.52	25.8 2.28	38.6^#^^ 2.10	45.7^#^^ 1.89*

^#^‘Top box’ percentage significantly different from *Control* at p** ≤** 0.05.

^^^‘Top box’ percentage significantly different from *Self-efficacy only* at p** ≤** 0.05.

*Mean score significantly different from *Control* at p **≤** 0.05.

### Perceived main message, emotional responses, defensive and unintended effects

Content analysis of answers to the question “What do you think is the main message in this advertisement? What is it telling people?” showed that the experimental concepts were effective at communicating at least one of the following main messages that directly aligned with the communication objectives (for a list of the range of communication objectives pertaining to the experimental concepts see Additional file 1). Up to two main messages were coded per participant. The perceived main messages for each of the experimental concepts and the percentage of participants who noted them were:

#### Self-efficacy only

• do not drink alcohol during pregnancy (71.2%);

• drink alcohol in moderation (11.4%); and

• no alcohol is the safest option during pregnancy (5.3%).

#### Threat only

• do not drink alcohol during pregnancy (55.4%);

• they (health authorities) aren’t sure how much alcohol will damage a baby so best not to drink at all (15.7%);

• no amount of alcohol is safe during pregnancy (10.8%); and

• you can damage your baby if you drink alcohol during pregnancy (8.4%).

#### Threat and self-efficacy

• do not drink alcohol during pregnancy (50.6%);

• they (health authorities) aren’t sure how much alcohol will damage a baby so best not to drink at all (16.0%);

• you can damage your baby if you drink alcohol during pregnancy (14.8%); and

• no amount of alcohol is safe during pregnancy (8.6%).

The three experimental concepts were rated well in terms of believability and relevance to women in general (Table 4) with over 50% of participants who saw them reporting that they were “very believable” and “very relevant to women in general”. The results show that *Threat and self-efficacy* was rated as interesting, convincing and as making the participant think about the topic in a new way by a greater percentage of participants than the other experimental concepts. All three experimental concepts rated well in providing important information.

**Table 4:** Message diagnostics (n = 354)

	***Control***	***Self-efficacy only***	***Threat only***	***Threat and self-efficacy***
	**n = 58**	**n = 132**	**n = 83**	**n = 81**
**Message diagnostics**	**t.b. %**$\bar{x}$	**t.b. %**$\bar{x}$	**t.b. %**$\bar{x}$	**t.b. %**$\bar{x}$
Believability	20.7 2.24	53.0^#^ 1.62*	55.4^#^ 1.64 *	54.3^#^ 1.53*
Relevance to women in general	24.1 2.07	58.3 1.63	56.6 1.53	61.7 1.53
Likeability	8.6 2.72	28.8^#^ 2.20*	22.9^#^ 2.42*	22.2^#^ 2.19*
	*yes%*	*yes%*	*yes%*	*yes%*
Interesting	51.7	74.2^#^	68.7^#^	77.8^#^
Convincing	56.9	74.2^#^	68.7^#^	84.0^#^~^
Makes me think about the topic in new way	25.9	40.2^#^	33.7	50.6^#~^
Provides important information	65.5	84.1^#^	92.8^#^^	91.4^#^

^#^’Top box’/affirmative answer percentage significantly different from *Control* at p **≤** 0.05.

^’Top box’/affirmative answer significantly different from *Self-efficacy only* at p **≤** 0.05.

~’Top box’/affirmative answer significantly different from *Threat only* at p **≤** 0.05.

*Mean score significantly different from *Control* at p **≤** 0.05.

Some distinct differences were found between the experimental concepts in the emotions they aroused for participants (Table 5). Those with threat appeals generated a small level of worry, anxiety and guilt, and these responses were not significantly attenuated by the addition of a self-efficacy message. Overall, the two threat concepts aroused more negative emotional responses (worry, anxiety, guilt, shame) and *Self-efficacy only* aroused more positive emotional responses (relief, happiness).The addition of a self-efficacy message to a threat appeal aroused more positive emotional responses of relief and happiness than the threat appeal alone.

**Table 5:** Emotional arousal (n = 354)

	***Control***	***Self-efficacy only***	***Threat only***	***Threat and self-efficacy***
	**n = 58**	**n = 132**	**n = 83**	**n = 81**
**Emotion**	**Yes %**	**Yes %**	**Yes %**	**Yes %**
Worried	17.2	12.9	27.7^#^^	23.5^^^
Anxious	10.3	9.8	20.5^#^^	17.3^^^
Guilty	29.3^^~*^	4.5	15.7^^^	13.6^^^
Regretful	17.2^^^~^*^	3.8	6.0	7.4
Ashamed	12.1^^*^	1.5	8.4^^^	6.2^^^
Surprised	13.8	26.5^#^	20.5	28.4^#^
Relieved	12.1	39.4^~^	26.5^#^	42.0~
Proud of myself	41.4	53.8^#^	56.6^#^	53.1^#^
Happy	48.3	77.3^#~*^	37.3	63.0^~^

^#^Affirmative answer percentage significantly different from *Control* at p **≤** 0.05.

^Affirmative answer percentage significantly different from *Self-efficacy only* at p **≤** 0.05.

~Affirmative answer percentage significantly different from *Threat only* at p **≤** 0.05.

^*^Affirmative answer percentage significantly different from *Threat and self-efficacy* at p **≤** 0.05.

Only a low proportion of participants experienced any defensive reactions as measured by the four responses relating to rejection of the message (Table 6). Furthermore, a very low percentage of participants indicated a potential for experiencing unintended effects following exposure to one of the experimental concepts. Importantly, there were no significant differences in this respect between women who saw an experimental concept and those who saw the control.

**Table 6:** Defensive responses and potential unintended effects (n = 354)

	***Control***	***Self-efficacy only***	***Threat only***	***Threat and self-efficacy***
	**n = 58**	**n = 132**	**n = 83**	**n = 81**
*Defensive responses*	*Yes %*	*Yes %*	*Yes %*	*Yes %*
I don’t want to think about what the advertisement is saying	8.6^^~^	3.0	3.6	8.6^^~^
The information in this advertisement is false	5.2^~^	3.0	1.2	1.2
The advertisement exaggerates the issue	12.1	7.6	13.3	9.9
The advertisement is misleading	6.9	6.1	4.8	3.7
*Potential unintended effects*				
Talk to a health professional about options for terminating the pregnancy	6.7	6.0	3.6	7.4
Drink the same amount of alcohol as usual for the rest of the pregnancy	1.7	0.0	1.2	0.0

Affirmative answer percentage significantly different from *Control* at p **≤** 0.05.

^Affirmative answer percentage significantly different from *Self-efficacy only* at p **≤** 0.05.

~Affirmative answer percentage significantly different from *Threat only* at p **≤** 0.05.

Multi-variable analyses showed no significant differences in the way in which participants with different characteristics responded to the concepts. Specifically, there were no significant differences in the responses to the two threat concepts compared to *Self-efficacy only* when viewed as a function of at-risk alcohol consumption, socioeconomic status, education and having borne children.

## Discussion

The relative effectiveness of a set of messages for a campaign aimed at promoting abstinence from alcohol during pregnancy among pregnant women and women of childbearing age was quantitatively tested against a control concept: one threat only concept; one self-efficacy only concept, and a threat plus self-efficacy concept. The concepts used were based on formative research with the target audience and theoretical constructs of behaviour change. Effectiveness was measured in terms of the impact on the audience’s intentions and confidence to abstain from or reduce alcohol intake in a current or future pregnancy. Several message diagnostics and potential to cause unintended effects were also measured.

Overall, the experimental concepts were effective at increasing women’s intentions to abstain from alcohol during pregnancy compared with the control. The two experimental threat concepts were significantly more effective on a broad range of measures than the control and the positive appeal (*Self-efficacy only*), and particularly with respect to behavioural intentions and confidence to modify behaviour. This result is consistent with research on the efficacy of fear (or threat) appeals [24,25] which shows that, provided the promoted response is under volitional control, the negative motivation of avoiding the threat is a powerful instigator of behaviour change. The quantitative results support the overall effectiveness of this message strategy and thus it is recommended that threat appeals, based on the negative motivation of avoiding poor health outcomes for the fetus, are considered for communications aimed at promoting abstinence from alcohol during pregnancy.

The potential risk of using threat appeals for the prevention of prenatal alcohol exposure has been raised [26,27]. One risk with threat-based messages is that they may be perceived by the audience to be sensationalising the severity of the consequences or over-stating the risks, and may thus be rejected and counter-argued [12]. However, the threat appeal concepts used in this study aroused few defensive responses or counter-arguments. Another risk that has been posited is that ‘hard-line’ or threat-based messages may lead a woman to consider a termination if she consumed alcohol during that pregnancy [28]. Again, the concepts used in this study avoided such reactions in that a similar and small proportion of women in each of the experimental groups and the control group reported that they may/ would talk to a health professional about options for terminating the pregnancy after having seen the advertisement. These findings reflect the utility of formative research is developing advertising concepts that not only motivate the desired behaviour but do so without stimulating unintended negative effects. The initial formative research provided insight into execution elements that were likely to be important to minimise unintended effects [18]. Specifically, the relevant findings were that if an honest and factual message is delivered by an expert and supportive source, along with an acknowledgement of the uncertainty surrounding risk to the fetus following low to moderate alcohol exposure in utero, then the message is likely to be persuasive as well as minimise counter-argument.

This study also assessed the differences between a threat appeal and a threat appeal combined with a self-efficacy message. This was done in acknowledgement of cognitive behavioural theories that argue that self-efficacy is an important component for instigating protection motivation [29] or a danger control process [30]. Generally, compared to the threat appeal alone the inclusion, of a self-efficacy message with the threat appeal did not increase behavioural intentions or participants’ confidence to modify their behaviour. However, the inclusion of a self-efficacy message with a threat appeal worked to lower the level of negative emotional arousal and increase positive emotional arousal.

Overall it is recommended that the concept combining threat with a self-efficacy message be developed for use in a campaign targeting alcohol use and pregnancy for two reasons. First, this concept had the greatest persuasive potential among the target audience compared with the other concepts. Second, compared with the threat only message*,* which was also shown to be persuasive, the combined threat/self-efficacy message is less likely to arouse negative emotions and lead to defensive motivation, rejection of the message and unintended effects.

### Limitations

A limitation of this study is that these messages were not tested with specific sub-groups of women who may respond differently or require different and more comprehensive strategies to support their abstinence from alcohol during pregnancy. The sample of participants was skewed towards an educated, middle socioeconomic-class sample of women, and the concepts were designed to target those who drink alcohol but generally not to excess. Research on women’s responses to warning messages about alcohol use during pregnancy in the USA showed that women who drank alcohol during pregnancy were much more likely to ignore warning messages, compared with those who abstained during pregnancy [31]. This suggests that the risk of message rejection may be greater among those women who are at higher risk of drinking alcohol during pregnancy, and different strategies may be required and should be evaluated for this group.

Another limitation is the set of concepts that were tested. As with any concepts there are many creative ways in which the content can be executed. Hence, there may be more effective ways to execute these messages, or communicate threat or promote self-efficacy.

## Conclusions

Education and awareness-raising campaigns represent one component of a comprehensive strategy to prevent alcohol exposed pregnancies and FASD. This study provides important insights into the elements that enhance the persuasiveness and effectiveness of messages aimed at preventing prenatal alcohol exposure and is one of the few studies in which such messages have been evaluated. The recommended concept, a threat appeal combined with a self-efficacy message has demonstrated potential to increase the target audiences’ intentions to abstain from alcohol during pregnancy with minimal negative consequences.

## Abbreviations

FASD: Fetal alcohol spectrum disorders.

## Competing interests

The authors declare that they have no competing interests.

## Authors’ contributions

All authors have read and approved the final manuscript. All authors originated the study. All authors contributed to conceptualizing ideas, interpreting findings and reviewing drafts of the article. KE France coordinated the research, lead the data collection, completed the analysis and first draft. RJ Donovan supervised the research design, data collection, quantitative analysis and preparation of results.

## Pre-publication history

The pre-publication history for this paper can be accessed here:

http://www.biomedcentral.com/1471-2458/14/30/prepub

## Supplementary Material

Additional file 1 Communication and modelling objectives. Click here for file
