# Supplementary material for: Messages that increase women’s intentions to abstain from alcohol during pregnancy: results from quantitative testing of advertising concepts
Source: BMC Public Health. 2014 Jan 13;14:30. doi: 10.1186/1471-2458-14-30 (PMC3903031; doi:10.1186/1471-2458-14-30)
Supplement: Additional file 1 — Communication and modelling objectives. [file 1471-2458-14-30-S1.docx]

# Appendix

## Appendix 1. Communication and modelling objectives

| *Communication objectives*  Create and reinforce the beliefs that:   - if you are pregnant you should reduce your alcohol intake, with abstinence as the primary goal; - alcohol consumption is something that (most) pregnant women can control and that reduction or abstinence from alcohol will support the health of the pregnancy and baby; - no alcohol during pregnancy is the safest option; - the risk to the fetus increases with increasing amount and increasing frequency and there is risk even when a woman is not ‘drunk’; - alcohol consumption is related to short-term and long-term negative consequences for the pregnancy and fetus; and - challenge the belief that ‘a couple of drinks every now and then’ are risk free.   *Modelling objectives*   - Align abstaining from alcohol to other positive behaviour changes for pregnancy such as ‘being on a health-kick’. - Show a significant person (i.e. partner or friend) supporting a woman to modify her alcohol consumption when pregnant. - Demonstrate a way of dealing with social situations when women want to abstain from alcohol but do not want others to know that they are trying to get pregnant or are pregnant. |
| --- |
